# Effects of sodium-glucose cotransporter 2 inhibitors on urinary excretion of intact and total angiotensinogen in patients with type 2 diabetes

**DOI:** 10.1136/jim-2017-000445

**Published:** 2017-06-08

**Authors:** Takuo Yoshimoto, Takayuki Furuki, Hiroyuki Kobori, Masaaki Miyakawa, Hitomi Imachi, Koji Murao, Akira Nishiyama

**Affiliations:** 1 Department of Endocrinology and Metabolism, Faculty of Medicine, Kagawa University, Miki - cho, Kagawa, Japan; 2 Department of Medicine, Hadanoeki – Minamiguchi Clinic, Hadano, Kanagawa, Japan; 3 Department of Pharmacology, Faculty of Medicine, Kagawa University, Miki - cho, Kagawa, Japan; 4 Department of Medicine, Miyakawa Clinic, Yokohama, Kanagawa, Japan

**Keywords:** SGLT2 inhibitor, urinary angiotensinogen, type 2 diabetes, blood pressure, urinary albumin

## Abstract

We conducted a descriptive case study to examine the effects of sodium-glucose cotransporter 2 (SGLT2) inhibitors on urinary angiotensinogen excretion, which represents the function of the intrarenal renin–angiotensin system, in patients with type 2 diabetes. An SGLT2 inhibitor (canagliflozin 100 mg/day, ipragliflozin 25 mg/day, dapagliflozin 5 mg/day, luseogliflozin 2.5 mg/day or tofogliflozin 20 mg/day) was administered for 1 month (n=9). ELISA kits were used to measure both urinary intact and total angiotensinogen levels. Treatment with SGLT2 inhibitors significantly decreased hemoglobin A1c, body weight, systolic blood pressure and diastolic blood pressure (8.5±1.3 to 7.5%±1.0%, 82.5±20.2 to 80.6±20.9 kg, 143±8 to 128±14 mm Hg, 78±10 to 67±9 mm Hg, p<0.05, respectively), while urinary albumin/creatinine ratio was not significantly changed (58.6±58.9 to 29.2±60.7 mg/g, p=0.16). Both total urinary angiotensinogen/creatinine ratio and intact urinary angiotensinogen/creatinine ratio tended to decrease after administration of SGLT2 inhibitors. However, these changes were not significant (p=0.19 and p=0.08, respectively). These data suggest that treatment with SGLT2 inhibitors does not activate the intrarenal renin–angiotensin system in patients with type 2 diabetes.

**Significance of this study**

**What is already known about this subject?**

- Urinary angiotensinogen has been proven to be a useful biomarker for monitoring the intrarenal renin–angiotensin system (RAS).
- Sodium-glucose cotransporter 2 (SGLT2) inhibitors may induce polyuria and dehydration. Dehydration activates the internal RAS.
- SGLT2 inhibitor increased the urinary angiotensinogen levels of patients with type 1 diabetes (not type 2 diabetes).

**What are the new findings?**

- SGLT2 inhibitor did not increase total urinary angiotensinogen levels of patients with type 2 diabetes (not type 1 diabetes).
- SGLT2 inhibitor did not increase intact urinary angiotensinogen levels of patients with type 2 diabetes.
- There are not many reports about the change of intact urinary angiotensinogen levels of patients with type 2 diabetes.

**How might these results change the focus of research or clinical practice?**

- SGLT2 inhibitors do not activate RAS in patients with type 2 diabetes.

## Introduction

Diabetes mellitus is a common disease worldwide,^1 2^ and results in many complications such as blindness, renal disease and myocardial dysfunction.^3^ Sodium-glucose cotransporter 2 (SGLT2) inhibitors increase excretion of glucose in the urine by inhibiting urinary reabsorption of glucose,^4^ independent of the effect of insulin.^5 6^ SGLT2 inhibitors block the reabsorption of glucose by SGLT2 at the S1 portion of the proximal tubule in the kidney.^7 8^ The American Diabetes Association has indicated that SGLT2 inhibitors are the second-line treatment after metformin and other oral hypoglycemic agents, basal insulin and glucagon-like peptide-1 receptor agonists.^9^ Clinical studies have shown that in patients with type 2 diabetes mellitus, administration of SGLT2 inhibitors resulted in sustained decreases in glycoproteins, hemoglobin A1c (HbA1c), body weight (BW), blood pressure (BP)^10^ and urine albumin/creatinine ratio.^11–13^ Recently, the beneficial role of SGLT2 inhibitors on cardiovascular and renal function was reported in the EMPA-REG OUTCOME trial.^14 15^

However, it has also been reported that SGLT2 inhibitors have many side effects, such as an increase in urine volume and polydipsia. In particular, Haneda *et al* 16 reported that SGLT2 inhibitors increased hematocrit and blood urea nitrogen/creatinine (BUN) ratio. These data suggest that treatment with SGLT2 inhibitors causes dehydration if water intake is not sufficient. In addition, it is feared that dehydration activates the renin–angiotensin system (RAS) in the kidney. Indeed, Cherney *et al* 17 reported that urinary total angiotensinogen excretion, which represents local RAS activity in the kidney,^18 19^ significantly increased owing to treatment with SGLT2 inhibitors in patients with type 1 diabetes mellitus. However, the effect of SGLT2 inhibitors on urinary angiotensinogen excretion in type 2 diabetes patients has not been investigated yet.

Therefore, we conducted a descriptive case study to examine the effects of SGLT2 inhibitors on urinary angiotensinogen in patients with type 2 diabetes.

## Methods

### Subjects

Studies were conducted on patients with type 2 diabetes whose blood glucose was inadequately controlled with diet/exercise therapy in combination with various oral antihyperglycemic drugs. Fourteen Japanese adults with type 2 diabetes (six men and eight women) aged 60.2±15.9 (31–81) were enrolled in this study. The study ran from September 1, 2014 to September 1, 2015 and was carried out by the Hadanoeki – Minamiguchi Clinic and Kagawa University Hospital. Study subjects had not been hospitalized for metabolic complications. The subjects had HbA1c ≧6.5% and creatinine clearance ≧60 mL/min. The exclusion criteria were insulin dependence, type 1 diabetes, pregnancy, severe anemia, macroalbuminuria, severe illness (including urinary tract infection), chronic liver disease, thyroid disease, adrenal disease and malignancy. The study was conducted in accordance with the ethical principles of the Declaration of Helsinki, and was approved by the ethics committee of Kagawa University (26-045). All patients gave written informed consent prior to participation.

### Study design

In this study, an SGLT2 inhibitor (canagliflozin 100 mg/day, ipragliflozin 25 mg/day, dapagliflozin 5 mg/day, luseogliflozin 2.5 mg/day or tofogliflozin 20 mg/day) was administered once daily for 1 month as an add-on therapy to other oral antihyperglycemic drugs (sulfonylurea, glinide, α-glucosidase inhibitor, biguanide, thiazolidines or dipeptidylpeptide-4 inhibitor), insulin and glucagon-like peptide-1. During the observational period, medications such as antihypertensive agents were not changed. No patient was treated with any diuretics. If BP was not well controlled, the study was stopped. If any adverse events occurred, for example severe skin disorders, liver failure, cardiovascular disorder and cerebrovascular disorder, these patients were excluded. We measured urinary angiotensinogen and urinary albumin before and after administering SGLT2 inhibitors (baseline and after 1 month). HbA1c, BW, systolic blood pressure (SBP) and diastolic blood pressure (DBP) were also measured.

### Assays

We measured urinary angiotensinogen levels by using a sandwich ELISA as described.^18 20^ HbA1c was determined using a latex Glycohemoglobin Standardization Program. Urinary albumin was measured using an assay kit (AutoWako Microalbumin; Wako Pure Chemical Industries, Osaka, Japan) and expressed as a protein to creatinine ratio in spot urine samples. HbA1c was measured by using high-performance liquid chromatography.

### Statistical analysis

Urinary angiotensinogen, urinary albumin, BW, HbA1c, SBP and DBP before and after treatment with SGLT2 inhibitors were expressed as mean±SD. Statistical analysis was performed with Student’s t-test, and statistical significance was defined as p<0.05.

## Results

The total number of samples in this study was nine (four men and five women). Five patients were excluded from the study due to discontinuation of the SGLT2 inhibitor. Treatment was terminated in five patients because one developed angina pectoris while the other four patients had poor compliance. The mean age of the group was 58.3±17.7 (31–81). Mean HbA1c was 8.5%±1.3%, mean BW was 82.5±20.2 kg, mean SBP was 143±8 mm Hg and mean DBP was 78±10 mm Hg.

Table 1 shows SGLT2 inhibitors significantly decreased BW, HbA1c, alanine transaminase (ALT), γ-glutamyltranspeptidase (γGTP) and total cholesterol. However, aspartate transaminase (AST) was not significantly changed. Creatinine did not change significantly, while BUN was slightly increased after administration of SGLT2 inhibitors. There was no significant relationship between changes in plasma ALT levels and those in intact or total urinary angiotensinogen/creatinine ratio before and after administration of an SGLT2 inhibitor (r=−0.42 and 0.16, respectively). Similarly, there was no significant relationship between changes in plasma AST levels and those in intact or total urinary angiotensinogen/creatinine ratio before and after administration of an SGLT2 inhibitor (r=0.10 and 0.04, respectively).

**Table 1:** Laboratory data before and after administration of an SGLT2 inhibitor

	Before	After	p
AST (U/L)	26±8	27±8	0.72
ALT (U/L)	38±21	34±20	0.012
γGTP (U/L)	68±54	42±26	0.013
BUN (mg/dL)	14.1±5.4	16.8±5.2	0.046
Cr (mg/dL)	0.69±0.17	0.74±0.18	0.16
T.Cho (mg/dL)	182±34	171±34	0.039
HDL-C (mg/dL)	51±17	50±17	0.37
TG (mg/dL)	171±87	159±97	0.57
Ht (%)	41±6	38±15	0.54
UA (mg/dL)	5.5±1.5	5.2±1.2	0.22
HbA1c (%)	8.5±1.3	7.5±1.0	0.0003
BW (kg)	82.5±20.2	80.6±20.9	0.013

Before and after administration of SGLT2 inhibitors.

ALT, alanine transaminase; AST, aspartate transaminase; BW, body weight; BUN, blood urea nitrogen/creatinine; γGTP, γ-glutamyltranspeptidase; Cr, creatinine; HbA1c, hemoglobin A1c; HDL-C, high-density lipoprotein - cholesterol; Ht, hematocrit; SGLT2, sodium-glucose co-transporter 2; T.Cho, total cholesterol; TG, triglyceride; UA, uric acid.

**Figure 1:**
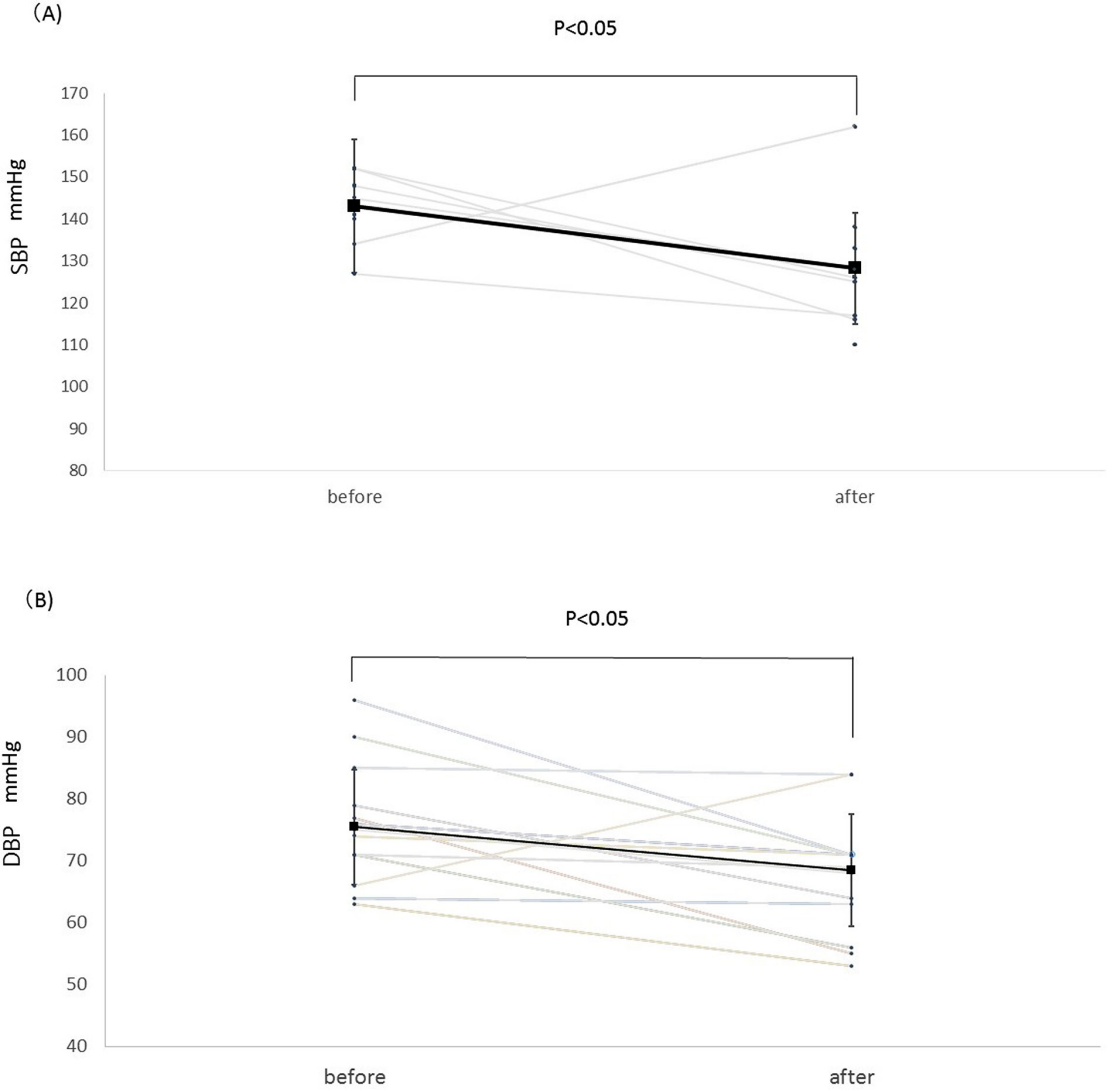
Change in SBP (A) and DBP (B) before and after administration of an SGLT2 inhibitor. Square points are shown as mean. SGLT2 inhibitors tended to decrease SBP and DBP, so these changes are statistically significant (p<0.05) before versus after treatment with an SGLT2 inhibitor. DBP, diastolic blood pressure; SBP, systolic blood pressure; SGLT2, sodium-glucose co-transporter 2.

Figure 1A shows the trend in SBP. Treatment with SGLT2 inhibitors significantly decreased SBP from 143±8 mm Hg to 128±14 mm Hg (p<0.05).

Similarly, SGLT2 inhibitors significantly decreased DBP from 78±10 mm Hg to 67±9 mm Hg (p<0.05, figure 1B).

**Figure 2:**
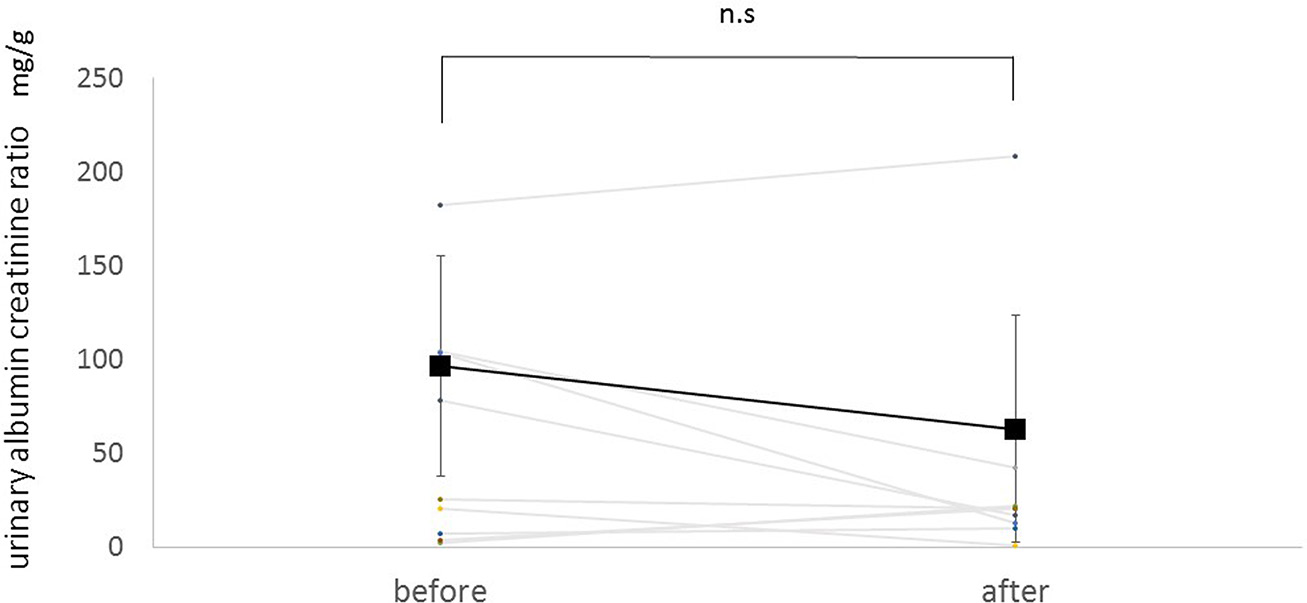
Change in urinary albumin/creatinine ratio before and after administration of an SGLT2 inhibitor. Square points are shown as mean. SGLT2 inhibitors tended to decrease urinary albumin/creatinine ratio, but these changes are not statistically significant (p=0.16). n.s., not significant; SGLT2, sodium-glucose co-transporter 2.

Figure 2 shows the trend in urinary albumin/creatinine ratio. Mean ratio at baseline was 58.6±58.9 mg/g. Treatment with SGLT2 inhibitors tended to decrease urinary albumin/creatinine ratio by 29.4 mg/g. However, these changes were not statistically significant (p=0.16).

**Figure 3:**
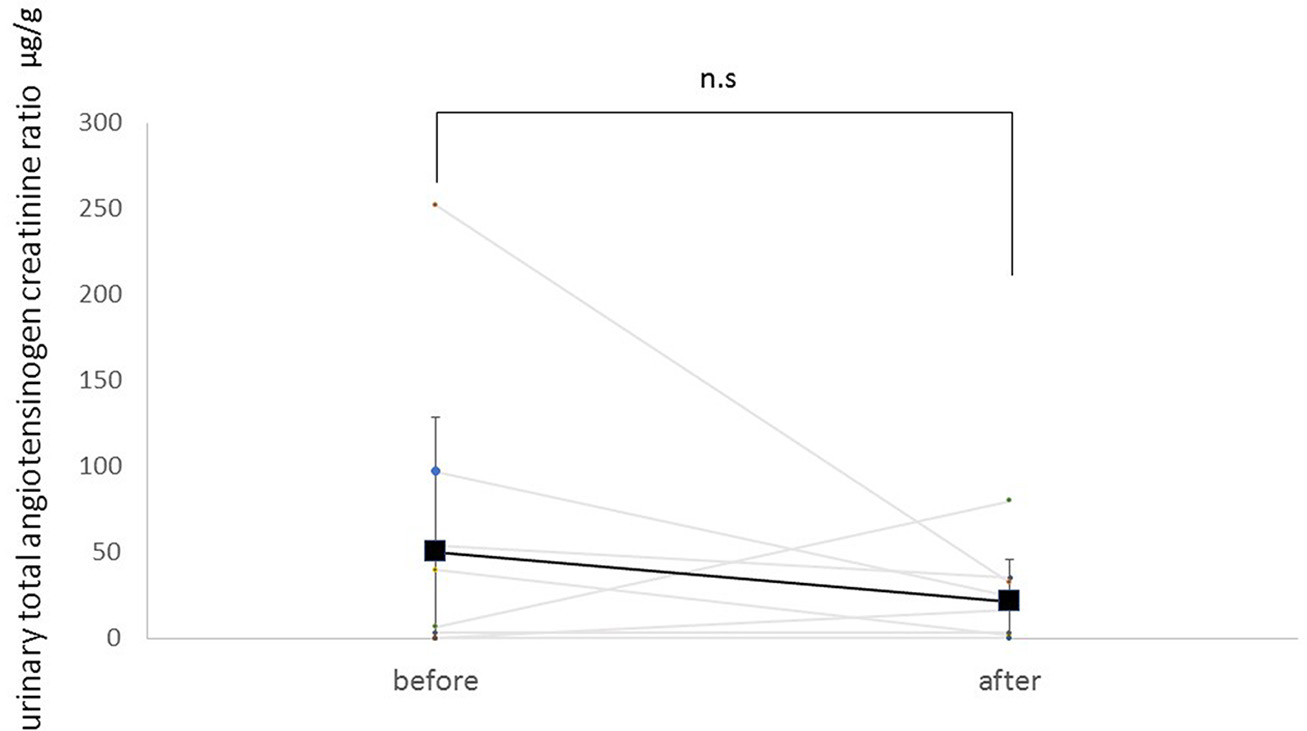
Change of urinary total angiotensinogen/creatinine ratio before and after administration of an SGLT2 inhibitor. Square points are shown as mean. SGLT2 inhibitors tended to decrease total urinary angiotensinogen/creatinine ratio, but these changes are not statistically significant (p=0.19). n.s., not significant; SGLT2, sodium-glucose co-transporter 2.

Figure 3 shows the trend in total urinary angiotensinogen/creatinine ratio. Mean ratio at baseline was 50.4±78.1 µg/g. Treatment with SGLT2 inhibitors tended to decrease total urinary angiotensinogen/creatinine ratio by 28.8 µg/g. However, these changes were not statistically significant (p=0.19).

**Figure 4:**
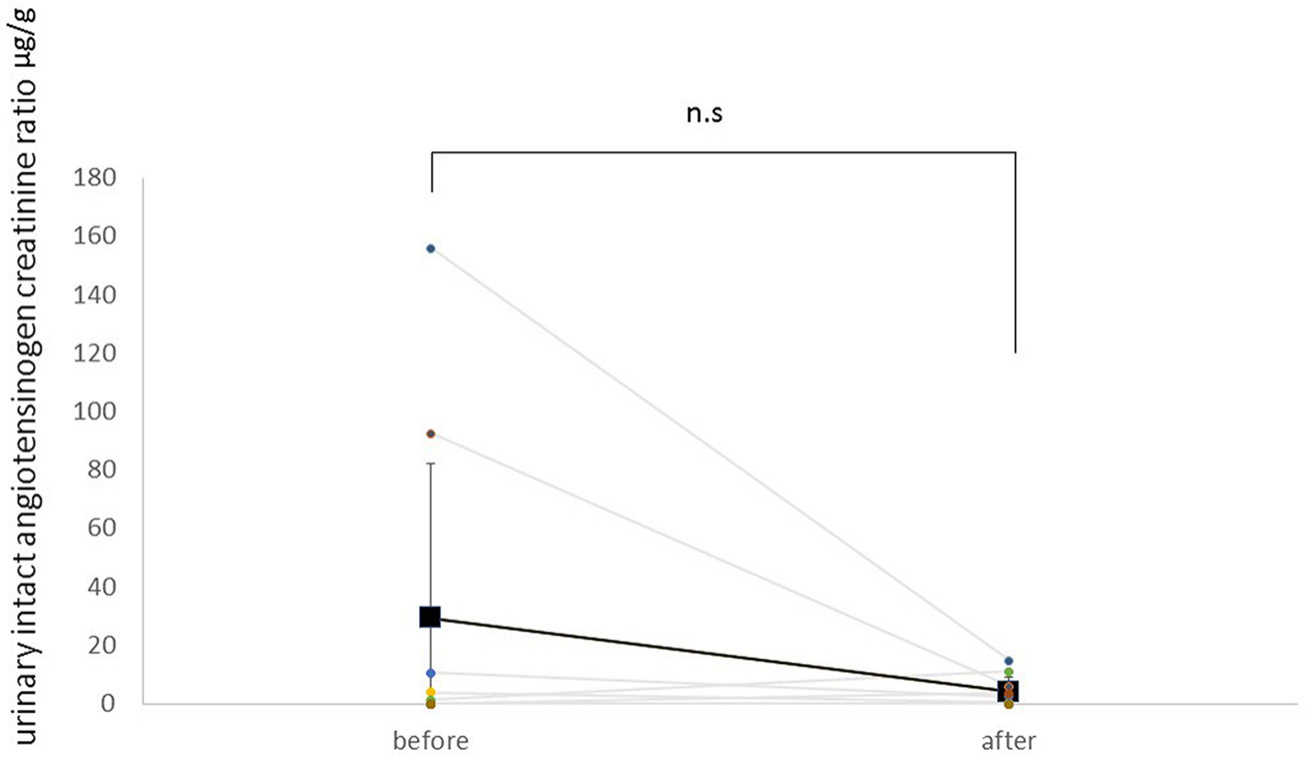
Change of urinary intact angiotensinogen/creatinine ratio before and after administration of an SGLT2 inhibitor. Square points are shown as mean. SGLT2 inhibitors tended to decrease intact urinary angiotensinogen/creatinine ratio, but these changes are not statistically significant (p=0.09). n.s., not significant; SGLT2, sodium-glucose co-transporter 2.

Figure 4 shows the trend in intact urinary angiotensinogen/creatinine ratio. Mean ratio at baseline was 29.4±53.0 µg/g. Treatment with SGLT2 inhibitors tended to decrease intact urinary angiotensinogen/creatinine ratio by 25.1 µg/g. However, these changes were not statistically significant (p=0.09).

## Discussion

Recent studies have shown that SGLT2 inhibitors increase plasma renin and total urinary angiotensinogen/creatinine ratio in patients with type 1 diabetes.^17 21^ These results suggest that treatment with an SGLT2 inhibitor stimulates RAS activity in the kidney. However, several preclinical studies have indicated that intrarenal RAS activity is much higher in type 2 diabetes than in type 1 diabetes.^18 22 23^ Therefore, we proposed a descriptive case study to examine the effect of SGLT2 inhibitors on urinary angiotensinogen excretion in patients with type 2 diabetes. We found that the baseline urinary total angiotensinogen/creatinine ratio in patients with type 2 diabetes was approximately 50 µg/g, which is much higher than in patients with type 1 diabetes,^24 25^ suggesting that intrarenal RAS is activated by augmentation of angiotensinogen. Our data also showed that urinary total angiotensinogen/creatinine ratio tended to be decreased by the administration of SGLT2 inhibitors, although the difference of total urinary angiotensinogen/creatinine ratio was not statistically significant. Collectively, these data support the hypothesis that SGLT2 inhibitors do not further activate intrarenal RAS in patients with type 2 diabetes whose basal RAS activation is already activated.

It is important to emphasize that urinary intact angiotensinogen was also measured in the present study. To the best of our knowledge, this is the first study involving the direct measurement of urinary intact angiotensinogen by ELISA in the human population. Intact urinary angiotensinogen is specific for angiotensinogen while total urinary angiotensinogen is associated with both intact angiotensinogen and des-angiotensin I angiotensinogen.^26^ Intact urinary angiotensinogen/creatinine ratio after administration of SGLT2 inhibitors was lower than the baseline urinary angiotensinogen/creatinine ratio, although this difference was not significant (p=0.09). Thus, the finding that administration of SGLT2 inhibitors did not change either total or intact angiotensinogen/creatinine ratio in the urine supports the hypothesis that intrarenal RAS is not activated by treatment with SGLT2 inhibitors in patients with type 2 diabetes.

In the present study, both total and intact angiotensinogen levels in the urine were measured. Since intact angiotensinogen does not contain cleaved fragments unlike total angiotensinogen,^26^ actual functional angiotensinogen concentration can be determined. The present study measured, for the first time, urinary intact angiotensinogen in humans. Our data showed that changes in urinary intact angiotensinogen excretion were similar to those in urinary total angiotensinogen in patients with type 2 diabetes. Future studies will be needed to determine the specific changes in urinary intact angiotensinogen.

In the present descriptive study, we examined the effects of several types of SGLT2 inhibitors on urinary angiotensinogen excretion. Clinical studies have shown that these SGLT2 inhibitors showed a similar reduction in HB1Ac.^16 27–31^ These data suggest that urinary excretion rates of glucose are also similar among all SGLT2 inhibitors; therefore, we speculate that renal effects of each SGLT2 inhibitors are also similar. Obviously, future studies are needed to determine whether all SGLT2 inhibitors similarly affect urinary angiotensinogen. In this study, some patients were treated with thiazolidines and other antihyperglycemic agents, in addition to an SGLT2 inhibitor. We did not observe any side effect such as edema and hypoglycemia in these patients before and after starting an SGLT2 inhibitor. Thus, it seems likely that the influence of these antihyperglycemic drugs on RAS is considered to be minimal. Plasma renin activity and aldosterone levels are useful markers to monitor systemic activities of the renin–angiotensin–aldosterone system.^32^ Further studies are needed to examine the effects of long treatment with an SGLT2 inhibitor on plasma renin activity and aldosterone levels in patients with type 2 diabetes. Further studies will also needed to compare the different effects of SGLT2 inhibitor on intrarenal RAS activity between types 1 and 2 diabetes with same protocols.

DeFronzo *et al* 22 showed that patients with macroalbuminuria demonstrated decreased urinary albumin levels, to levels consistent with microalbuminuria, following treatment with SGLT2 inhibitors. Similarly, treatment with SGLT2 inhibitors decreased urinary albumin excretion in patients with type 2 diabetes who showed both microalbuminuria and macroalbuminuria.^11^ In the present descriptive case study, SGLT2 inhibitors tended to decrease urinary albumin/creatinine ratio, but these changes were not statistically significant (p=0.16). A failure to observe a significant reduction in albuminuria may be due to small sample size and/or short observation period. In this study, almost all patients with diabetes showed normoalbuminuria and the urinary albumin/creatinine ratio was not changed. These data are in agreement with those in the subanalysis of the EMPA-REG OUTCOME study, showing that SGLT2 inhibitors did not prevent the occurrence of microalbuminuria.^15^ Since we measured urinary albumin in spot urine samples, further studies with pooled urine samples should be performed in larger sample size with a longer observation period.

In agreement with other clinical studies,^11 23^ the present study showed that SGLT2 inhibitors reduce both BP and HbA1c in patients with type 2 diabetes. We also observed that treatment with SGLT2 inhibitors significantly decreased both ALT and γGTP. Ohki *et al* 33 reported a significant reduction in serum ALT levels following treatment with SGLT2 inhibitors in Japanese, type 2 diabetic patients with non-alcoholic fatty liver disease. Interestingly, they also showed that SGLT2 inhibitors improved the index of Fibrosis-4, which is a marker for predicting the fibrous change of fatty liver.^34^ In this study, treatment with an SGLT2 inhibitor significantly decreased plasma ALT and AST levels; however, these changes were not significantly correlated with those in urinary intact or total angiotensinogen/creatinine ratio.

In conclusion, the present descriptive case study has indicated that treatment with SGLT2 inhibitors does not activate intrarenal RAS in patients with type 2 diabetes. Further clinical studies using larger sample sizes and longer observation periods are required to corroborate this conclusion.
